# Resistin and adenylyl cyclase-associated protein 1 (CAP1) regulate the expression of genes related to insulin resistance in BNL CL.2 mouse liver cells

**DOI:** 10.1016/j.dib.2019.104112

**Published:** 2019-06-08

**Authors:** Dimiter Avtanski, Karin Chen, Leonid Poretsky

**Affiliations:** Gerald J. Friedman Diabetes Institute at Lenox Hill Hospital, Northwell Health, New York, NY, USA

**Keywords:** Resistin, Adenylyl cyclase-associated protein 1 (CAP1), Insulin resistance, BNL CL.2 cells

## Abstract

Resistin is an adipokine produced in white adipose tissue that is thought to modulate insulin sensitivity in peripheral tissues (such as liver, skeletal muscle or adipose tissue). Human and murine resistin molecules share only about 60% sequence homology. [1] Contrary to humans, in which resistin is secreted mostly by macrophages, Park and Ahima 2013 resistin in rodents is produced primarily by the mature adipocytes of the white adipose tissue. Although resistin can bind to toll-like receptor 4 (TLF4) activating proinflammatory responses in human and rodents, [3], [4], [5], [6], [7], [8] the inflammatory actions of resistin in human monocytes were found to be mediated by resistin binding to adenylyl cyclase-associated protein 1 (CAP1). [9] In this study, we aimed to investigate the *in vitro* effects of resistin on the expression of various genes related to insulin resistance in mouse liver cells. Using BNL CL.2 cells, we investigated the effect of resistin in untransfected or CAP1 siRNA-transfected cells on the expression of 84 key genes involved in insulin resistance.

**Table:** 

Subject area	Biology
More specific subject area	Inflammation
Type of data	Graphs, figures
How data was acquired	qRT-PCR array, QuantStudio 3 (Life Technologies)
Data format	Analyzed
Experimental factors	BNL CL.2 cells treated with resistin in the presence or absence of CAP1 siRNA transfection
Experimental features	Untransfected or CAP1 siRNA-transfected BNL CL.2 cells were treated with resistin, and mRNA expression levels of 84 genes related to insulin resistance were measured by using qRT-PCR array.
Data source location	New York, New York, USA
Data accessibility	Data is with this article
Related research article	Lee S, Lee H–C, Kwon Y–W, Lee SE, Cho Y, Kim J et al. Adenylyl cyclase-associated protein 1 is a receptor for human resistin and mediates inflammatory actions of human monocytes. Cell Metab. 2014 Mar 4; 19(3):484–97

**Table:** 

**Value of the data** • The role of murine resistin in mediating insulin sensitivity of the peripheral tissues and adenylyl cyclase-associated protein 1 (CAP1) as potential resistin receptor is not yet well understood. • We used BNL CL.2 mouse liver cells that were treated with resistin in the presence or absence of CAP1 siRNA transfection. • We found that resistin modulates gene expression of several genes related to insulin resistance, and the effect of some of these genes is modulated by CAP1.

## 1 Data

### 1.1 Optimization of CAP1 siRNA transfections

Our optimization experiments (data not shown) demonstrated that there was no statistically significant difference in CAP1 mRNA expression levels between untransfected and siRNA negative control-transfected cells. There was a significant decrease (86%) in CAP1 siRNA levels in CAP1 siRNA-transfected cells, compared to untransfected controls. There was no statistically significant difference in the cell viability between all of the treatment conditions.

#### 1.2 Effect of resistin on the expression of genes related to insulin resistance in BNL CL.2 cells

**Fig. 1:**
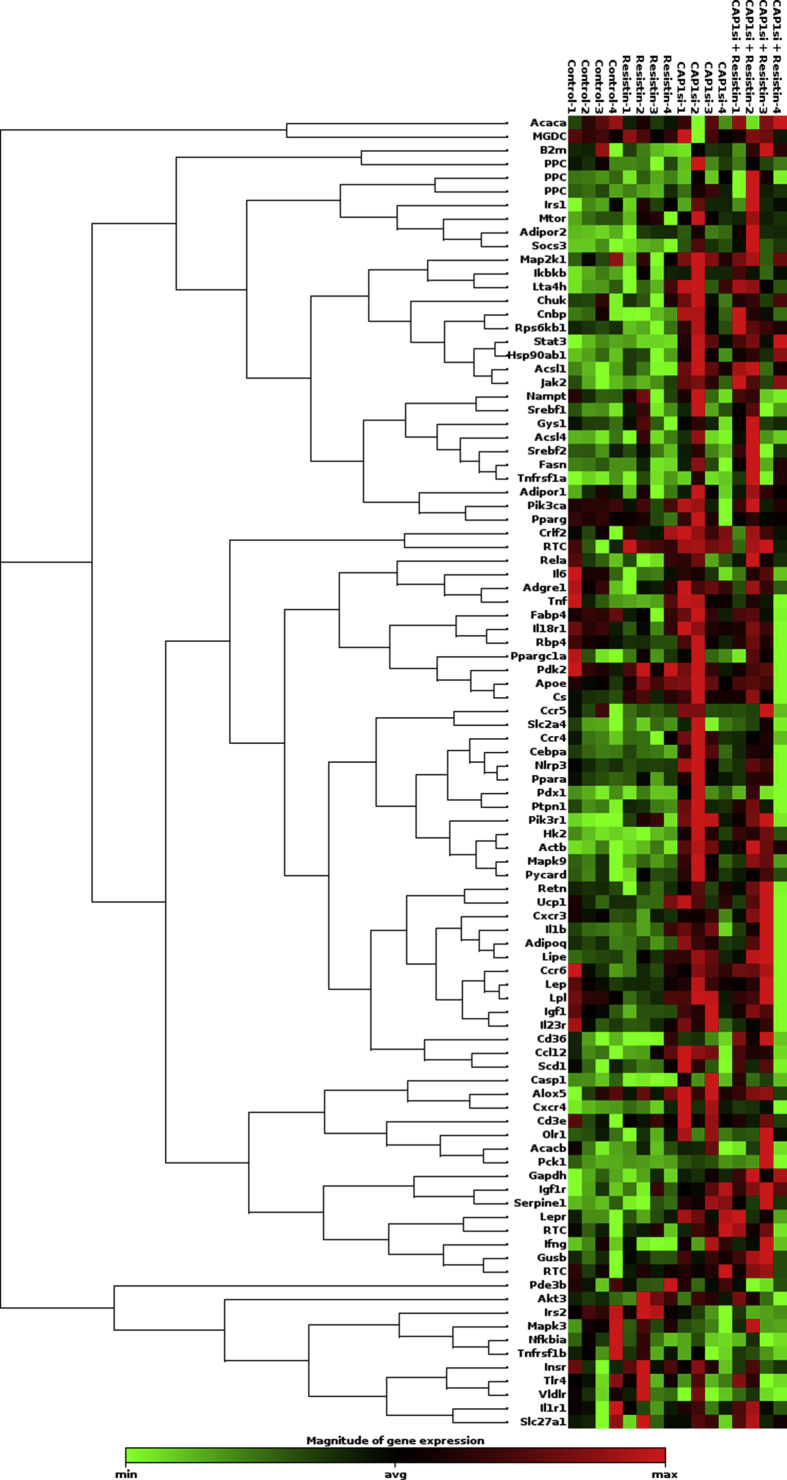
**Gene expression–clustergram.** Clustergram of the entire dataset displaying a heat map with dendrograms indicating co-regulated genes across groups or individual samples.

Using quantitative RT-PCR array, we measured the expression levels of 84 key genes involved in the mechanisms behind type 2 *diabetes mellitus* (T2DM) in adipose tissue (the full list of genes is presented in Table 1, and list of genes grouped by function is provided in Table 2) in BNL CL.2 cells in the presence or absence of resistin (25 ng/ml for 24 hours) (Fig. 1).

**Table 1:** **List of genes examined.** Table list of all genes measured by the PCR array, including the NCBI reference sequence database (RefSeq), gene abbreviations, full names and/or synonyms.

RefSeq	Abbreviation	Full Name
NM_133360	Acaca	Acetyl-Coenzyme A carboxylase alpha
NM_133904	Acacb	Acetyl-Coenzyme A carboxylase beta
NM_007981	Acsl1	Acyl-CoA synthetase long-chain family member 1
NM_019477	Acsl4	Acyl-CoA synthetase long-chain family member 4
NM_009605	Adipoq	Adiponectin, C1Q and collagen domain containing
NM_028320	Adipor1	Adiponectin receptor 1
NM_197985	Adipor2	Adiponectin receptor 2
NM_011785	Akt3	Thymoma viral proto-oncogene 3
NM_009662	Alox5	Arachidonate 5-lipoxygenase
NM_009696	Apoe	Apolipoprotein E
NM_009807	Casp1	Caspase 1
NM_011331	Ccl12	Chemokine (C–C motif) ligand 12
NM_009916	Ccr4	Chemokine (C–C motif) receptor 4
NM_009917	Ccr5	Chemokine (C–C motif) receptor 5
NM_009835	Ccr6	Chemokine (C–C motif) receptor 6
NM_007643	Cd36	CD36 antigen
NM_007648	Cd3e	CD3 antigen, epsilon polypeptide
NM_007678	Cebpa	CCAAT/enhancer binding protein (C/EBP), alpha
NM_007700	Chuk	Conserved helix-loop-helix ubiquitous kinase
NM_013493	Cnbp	Cellular nucleic acid binding protein
NM_016715	Crlf2	Cytokine receptor-like factor 2
NM_026444	Cs	Citrate synthase
NM_009910	Cxcr3	Chemokine (C-X-C motif) receptor 3
NM_009911	Cxcr4	Chemokine (C-X-C motif) receptor 4
NM_010130	Adgre1	EGF-like module containing, mucin-like, hormone receptor-like sequence 1
NM_024406	Fabp4	Fatty acid binding protein 4, adipocyte
NM_007988	Fasn	Fatty acid synthase
NM_030678	Gys1	Glycogen synthase 1, muscle
NM_013820	Hk2	Hexokinase 2
NM_008337	Ifng	Interferon gamma
NM_010512	Igf1	Insulin-like growth factor 1
NM_010513	Igf1r	Insulin-like growth factor I receptor
NM_010546	Ikbkb	Inhibitor of kappaB kinase beta
NM_008365	Il18r1	Interleukin 18 receptor 1
NM_008361	Il1b	Interleukin 1 beta
NM_008362	Il1r1	Interleukin 1 receptor, type I
NM_144548	Il23r	Interleukin 23 receptor
NM_001314054	Il6	Interleukin 6
NM_010568	Insr	Insulin receptor
NM_010570	Irs1	Insulin receptor substrate 1
NM_001081212	Irs2	Insulin receptor substrate 2
NM_008413	Jak2	Janus kinase 2
NM_008493	Lep	Leptin
NM_010704	Lepr	Leptin receptor
NM_010719	Lipe	Lipase, hormone sensitive
NM_008509	Lpl	Lipoprotein lipase
NM_008517	Lta4h	Leukotriene A4 hydrolase
NM_008927	Map2k1	Mitogen-activated protein kinase kinase 1
NM_011952	Mapk3	Mitogen-activated protein kinase 3
NM_016961	Mapk9	Mitogen-activated protein kinase 9
NM_020009	Mtor	Mechanistic target of rapamycin (serine/threonine kinase)
NM_021524	Nampt	Nicotinamide phosphoribosyltransferase
NM_010907	Nfkbia	Nuclear factor of kappa light polypeptide gene enhancer in B-cells inhibitor, alpha
NM_145827	Nlrp3	NLR family, pyrin domain containing 3
NM_138648	Olr1	Oxidized low density lipoprotein (lectin-like) receptor 1
NM_011044	Pck1	Phosphoenolpyruvate carboxykinase 1, cytosolic
NM_011055	Pde3b	Phosphodiesterase 3B, cGMP-inhibited
NM_133667	Pdk2	Pyruvate dehydrogenase kinase, isoenzyme 2
NM_008814	Pdx1	Pancreatic and duodenal homeobox 1
NM_008839	Pik3ca	Phosphatidylinositol 3-kinase, catalytic, alpha polypeptide
NM_001024955	Pik3r1	Phosphatidylinositol 3-kinase, regulatory subunit, polypeptide 1 (p85 alpha)
NM_011144	Ppara	Peroxisome proliferator activated receptor alpha
NM_011146	Pparg	Peroxisome proliferator activated receptor gamma
NM_008904	Ppargc1a	Peroxisome proliferative activated receptor, gamma, coactivator 1 alpha
NM_011201	Ptpn1	Protein tyrosine phosphatase, non-receptor type 1
NM_023258	Pycard	PYD and CARD domain containing
NM_011255	Rbp4	Retinol binding protein 4, plasma
NM_009045	Rela	V-rel reticuloendotheliosis viral oncogene homolog A (avian)
NM_022984	Retn	Resistin
NM_028259	Rps6kb1	Ribosomal protein S6 kinase, polypeptide 1
NM_009127	Scd1	Stearoyl-Coenzyme A desaturase 1
NM_008871	Serpine1	Serine (or cysteine) peptidase inhibitor, clade E, member 1
NM_011977	Slc27a1	Solute carrier family 27 (fatty acid transporter), member 1
NM_009204	Slc2a4	Solute carrier family 2 (facilitated glucose transporter), member 4
NM_007707	Socs3	Suppressor of cytokine signaling 3
NM_011480	Srebf1	Sterol regulatory element binding transcription factor 1
NM_033218	Srebf2	Sterol regulatory element binding factor 2
NM_011486	Stat3	Signal transducer and activator of transcription 3
NM_021297	Tlr4	Toll-like receptor 4
NM_013693	Tnf	Tumor necrosis factor
NM_011609	Tnfrsf1a	Tumor necrosis factor receptor superfamily, member 1a
NM_011610	Tnfrsf1b	Tumor necrosis factor receptor superfamily, member 1b
NM_009463	Ucp1	Uncoupling protein 1 (mitochondrial, proton carrier)
NM_013703	Vldlr	Very low density lipoprotein receptor
NM_007393	Actb	Actin, beta
NM_009735	B2m	Beta-2 microglobulin
NM_010368	Gusb	Glucuronidase, beta
NM_008084	Gapdh	Glyceraldehyde-3-phosphate dehydrogenase
NM_008302	Hsp90ab1	Heat shock protein 90 alpha (cytosolic), class B member 1
SA_00106	MGDC	Mouse Genomic DNA Contamination
SA_00104	RTC	Reverse Transcription Control
SA_00104	RTC	Reverse Transcription Control
SA_00104	RTC	Reverse Transcription Control
SA_00103	PPC	Positive PCR Control
SA_00103	PPC	Positive PCR Control
SA_00103	PPC	Positive PCR Control

**Table 2:** **Genes by function.** Table lists all of the genes examined, separated by function.

Function	Gene
Insulin signaling	Akt3
	Gys1
	Igf1
	Igf1r
	Ikbkb(IKK2)
	Insr
	Irs1
	Irs2
	Map2k1(Mek1)
	Mapk3(Erk1)
	Mapk9(Jnk2)
	Mtor
	Pde3b
	Pik3ca(p110-alpha)
	Pik3r1(PI3KA, p85alpha)
	Ppargc1a(Pgc-1alpha, Ppargc1)
	Ptpn1(Ptp1b, Ptp)
	Rps6kb1
	Slc2a4(Glut4)
	Socs3
	Srebf1
Non-insulin dependent diabetes mellitus	Adipoq(Acrp30)
	Hk2
	Ikbkb(IKKbeta, IKK2)
	Insr
	Irs1
	Irs2
	Mapk3(Erk1)
	Mapk9(Jnk2)
	Mtor
	Pdx1(Ipf1)
	Pik3ca(p110-alpha)
	Pik3r1(PI3KA, p85-alpha)
	Slc2a4(Glut4)
	Socs3
	Tnf
Adipokine signaling	
*Adipokines*	Adipoq(Acrp30)
	Il6
	Lep
	Nampt
	Retn
	Serpine1(PAI-1)
	Tnf
*Receptors & transporters*	Adipor1
	Adipor2
	Cd36
	Lepr
	Slc2a4(Glut4)
	Tnfrsf1a(Tnfr1)
	Tnfrsf1b
*Signaling downstream of adipokines*	Akt3
	Chuk(Ikbka, Ikka)
	Ikbkb(IKK2)
	Irs1
	Irs2
	Jak2
	Mapk9(Jnk2)
	Mtor
	Nfkbia(Iκb-alpha, Mad3)
	Ppara
	Ppargc1a(Ppargc1)
	Rela
	Socs3
	Stat3
Innate immunity	Casp1(ICE)
	Chuk(Ikbka, Ikka)
	Ikbkb(IKK2)
	Irs1
	Irs2
	Nlrp3
	Nfkbia(Iκb-alpha, Mad3)
	Pycard(Tms1, Asc)
	Rela
	Tlr4
Inflammation	Alox5
	Casp1(Ice)
	Ccl12(MCP-5, Scya12)
	Ccr4
	Ccr5
	Chuk(Ikbka, Ikka)
	Cxcr4
	Ifng
	Ikbkb(IKK2)
	Il1b
	Il23r
	Il6
	Lta4h
	Nlrp3
	Olr1
	Pycard(Tms1, Asc)
	Rela
	Tnf
	Tnfrsf1a(Tnfr1)
	Tnfrsf1b
Apoptosis	Pparg
	Serpine1PAI-1)
	Tnf
	Casp1(Ice)
	Ikbkb(IKK2)
	Irs2
	Mapk9(Jnk2)
	Nfkbia(Iκb-alpha, Mad3)
	Nlrp3
	Pycard(Tms1, Asc)
	Rela
	Tnfrsf1a (Tnfr1)
	Jak2
	Pik3ca (p110-alpha)
	Socs3
	Rps6kb1
	Tnfrsf1b
Metabolic pathways	
*Carbohydrate metabolism*	Cs
	Gys1
	Hk2
	Pck1
	Pdk2
*Lipid metabolism*	Acaca
	Acacb
	Acsl1
	Acsl4
	Apoe
	Cebpa
	Cnbp
	Fabp4
	Fasn
	Lepr
	Lipe
	Lpl
	Ppara
	Pparg
	Ppargc1a(Ppargc1)
	Scd1
	Srebf1
	Srebf2
*Metabolite transport*	Apoe
	Cd36
	Fabp4
	Rbp4
	Slc2a4(Glut4)
	Slc27a1
	Ucp1
	Vldlr
Infiltrating leukocyte markers	
*Macrophages*	Ccr5
	Cxcr4
	Adgre1
*Th1 cells*	Ccr5
	Cd3e
	Cxcr3
	Il18r1
*Th2 cells*	Ccr4
	Cd3e
	Crlf2 (Tslpr)
	Il1r1
*Th17 cells*	Ccr6
	Cd3e
	Il23r

**Fig. 2:**
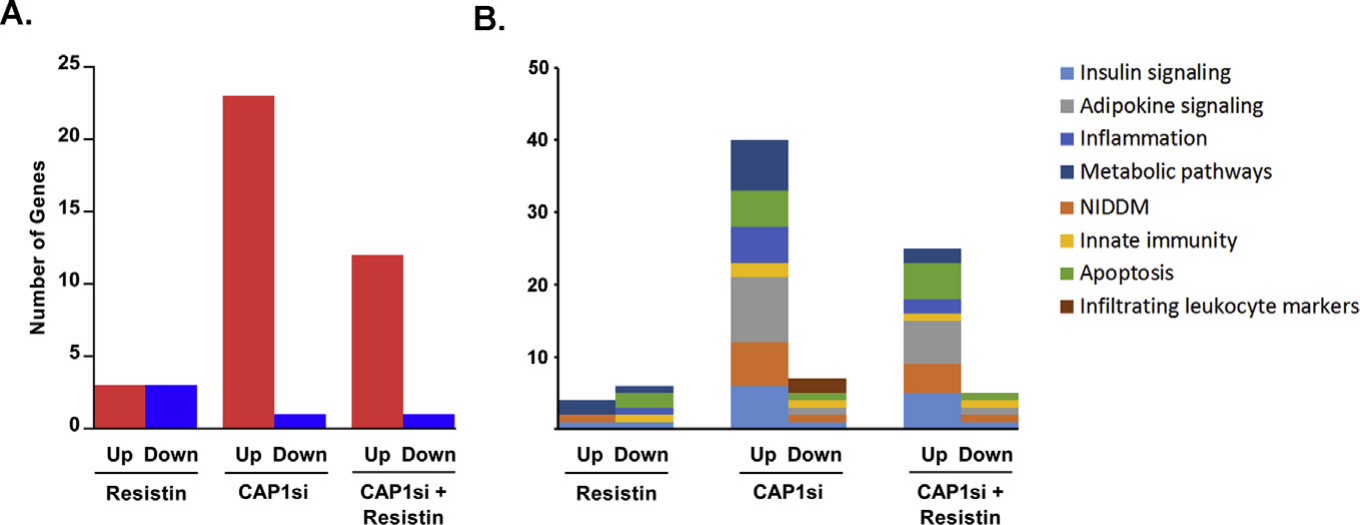
**Number of genes significantly affected by treatment. A.** The graph represents the number of statistically significant (p < 0.05) up- or down-regulated genes. **B.** Graph represents genes significantly up- or down-regulated within each treatment group, grouped by function.

Resistin treatment resulted in statistically significant change in the expression of 6 genes (Table 3). Of these genes, 3 were significantly upregulated (Apoe, Cs, and Pik3rl) and 3 downregulated (Casp1, Rbp4, and Rps6kb1) (Fig. 2A). Functionally, these genes participate in metabolic pathways (Apoe, Cs, and Rbp4), insulin signaling (Pik3r1 and Rps6kb1), T2DM (Pik3r1), innate immunity (Casp1), inflammation (Casp1), and apoptosis (Casp1 and Rps6kb1) (Fig. 2B).

**Table 3:** **Fold regulation of gene expression (treatment *vs.* control).** Fold change of gene expression of each treatment *vs.* control. p values <= 0.05 are marked in bold.

Gene	Resistin		CAP1 siRNA		CAP1 siRNA + Resistin	
	Fold Regulation	p-value	Fold Regulation	p-value	Fold Regulation	p-value
Acaca	−1.14	0.169661	−1.22	0.290632	1.00	0.878421
Acacb	1.01	0.906748	1.09	0.438927	1.06	0.643704
Acsl1	1.02	0.724041	1.44	**0.000491**	1.36	**0.018940**
Acsl4	1.04	0.542086	1.08	0.356823	1.12	0.206335
Adipoq	−1.10	0.173396	1.32	**0.041017**	1.15	0.401499
Adipor1	−1.04	0.329231	1.02	0.640683	1.06	0.146953
Adipor2	1.13	0.113931	1.17	**0.028059**	1.34	**0.016324**
Akt3	1.07	0.126858	1.03	0.256181	1.01	0.782413
Alox5	1.42	0.364008	1.75	0.099439	1.51	0.263775
Apoe	1.55	**0.022577**	1.29	**0.046392**	−1.02	0.812274
Casp1	−1.89	**0.003612**	1.41	0.224487	1.24	0.252274
Ccl12	1.22	0.306190	1.51	0.099450	1.33	0.142705
Ccr4	1.00	0.925798	1.52	**0.021117**	1.17	0.271836
Ccr5	−1.05	0.612433	1.27	0.357504	1.07	0.791426
Ccr6	−1.07	0.570025	1.14	0.358616	1.07	0.661161
Cd36	−1.08	0.979498	1.17	0.480636	1.74	0.080825
Cd3e	1.30	0.367086	1.55	0.122220	1.28	0.416990
Cebpa	−1.01	0.554566	1.16	0.045397	1.05	0.407643
Chuk	−1.00	0.923392	1.07	0.230993	1.06	0.155313
Cnbp	−1.08	0.148730	1.15	0.098214	1.12	0.158070
Crlf2	1.13	0.152933	1.24	**0.010318**	1.10	0.290357
Cs	1.49	**0.025871**	1.28	**0.023492**	1.06	0.583900
Cxcr3	−1.16	0.143245	1.02	0.813042	1.05	0.617630
Cxcr4	1.14	0.144379	1.66	**0.013166**	1.20	0.141848
Adgre1	−1.15	0.431959	1.20	0.273281	−1.00	0.936820
Fabp4	−1.21	0.123579	1.02	0.702554	−1.14	0.343194
Fasn	−1.02	0.563222	1.05	0.370487	1.11	0.102888
Gys1	1.00	0.891518	1.01	0.721490	1.05	0.204388
Hk2	−1.02	0.556124	1.39	**0.009742**	1.37	**0.004545**
Ifng	−1.42	0.518183	1.76	0.147576	1.54	0.290151
Igf1	−1.20	0.063415	1.12	0.298831	−1.00	0.909747
Igf1r	1.03	0.376609	1.12	**0.009107**	1.15	**0.000795**
Ikbkb	1.02	0.477848	1.10	**0.031863**	1.08	**0.013828**
Il18r1	−1.14	0.127650	1.07	0.378861	−1.08	0.592830
Il1b	1.06	0.555982	1.26	0.122392	1.16	0.340112
Il1r1	−1.06	0.560278	1.01	0.957418	1.07	0.683408
Il23r	−1.13	0.426607	1.16	0.476194	−1.18	0.546692
Il6	−1.46	0.078072	−1.21	0.351666	−1.20	0.426350
Insr	1.11	0.130969	1.04	0.591773	1.02	0.833929
Irs1	1.12	0.305636	1.15	0.219709	1.20	0.177262
Irs2	1.01	0.775771	−1.13	0.017086	−1.14	**0.003924**
Jak2	1.03	0.424419	1.23	**0.013603**	1.27	**0.009525**
Lep	−1.08	0.361070	1.16	0.211892	−1.04	0.971677
Lepr	1.11	0.377068	1.59	**0.005756**	1.30	0.159982
Lipe	1.06	0.206055	1.17	**0.012267**	1.12	0.252451
Lpl	−1.21	0.124427	1.10	0.365642	−1.10	0.808003
Lta4h	1.02	0.531694	1.13	**0.016815**	1.11	**0.022074**
Map2k1	−1.06	0.445888	1.10	0.078819	1.06	0.321980
Mapk3	−1.02	0.680433	−1.09	0.096201	−1.04	0.580686
Mapk9	−1.00	0.917290	1.16	0.065311	1.13	0.132737
Mtor	1.03	0.684468	1.24	**0.049218**	1.19	0.080924
Nampt	1.01	0.846387	1.02	0.732144	−1.02	0.896754
Nfkbia	−1.09	0.318262	−1.16	0.065727	−1.17	0.069963
Nlrp3	−1.06	0.126789	1.24	0.126506	1.01	0.762575
Olr1	−1.38	0.504119	1.35	0.272188	1.33	0.287191
Pck1	−1.05	0.568571	1.31	0.077792	1.16	0.490821
Pde3b	1.02	0.825368	1.03	0.787814	−1.15	0.190104
Pdk2	1.14	0.394247	1.06	0.729603	−1.23	0.473372
Pdx1	−1.01	0.710029	1.24	0.100216	1.04	0.657469
Pik3ca	−1.03	0.399336	−1.03	0.782751	1.01	0.744423
Pik3r1	1.52	**0.033282**	1.25	**0.000901**	1.18	**0.034911**
Ppara	−1.02	0.792466	1.21	0.080647	1.02	0.722852
Pparg	−1.08	0.093212	−1.03	0.865268	−1.03	0.216785
Ppargc1a	1.01	0.998904	1.07	0.594209	1.00	0.998558
Ptpn1	1.07	0.237874	1.29	**0.032159**	1.16	0.189144
Pycard	1.08	0.551026	1.42	**0.036940**	1.29	0.054659
Rbp4	−1.56	**0.007272**	1.06	0.482300	−1.07	0.751105
Rela	−1.04	0.598118	1.03	0.646466	1.07	0.279014
Retn	−1.10	0.204038	1.11	0.099451	1.10	0.388516
Rps6kb1	−1.58	**0.002976**	1.06	0.144979	1.11	**0.004902**
Scd1	1.05	0.821963	1.17	0.461504	1.18	0.410142
Serpine1	−1.03	0.820937	1.56	**0.001029**	1.37	**0.043504**
Slc27a1	1.01	0.929373	1.04	0.805979	1.13	0.317341
Slc2a4	1.04	0.761240	1.24	0.303759	1.15	0.331140
Socs3	−1.01	0.829376	1.27	**0.021130**	1.34	**0.039503**
Srebf1	1.10	0.340675	1.18	0.186347	1.11	0.348476
Srebf2	−1.01	0.876772	1.02	0.732351	1.12	0.368694
Stat3	−1.01	0.653690	1.27	0.002095	1.29	0.001880
Tlr4	1.03	0.737080	−1.05	0.492373	−1.03	0.761516
Tnf	−1.22	0.480161	1.33	0.255783	−1.14	0.607219
Tnfrsf1a	1.02	0.425885	1.05	0.359944	1.15	0.089583
Tnfrsf1b	−1.06	0.567979	−1.22	0.139907	−1.17	0.161986
Ucp1	1.01	0.881083	1.20	0.097531	1.05	0.631065
Vldlr	1.04	0.699680	−1.11	0.501624	−1.13	0.174714
Actb	1.01	0.777600	1.33	**0.001409**	1.33	**0.001148**
B2m	−1.06	0.258822	−1.02	0.642430	1.05	0.470054
Gapdh	1.01	0.814693	1.21	**0.004777**	1.45	**0.001734**
Gusb	1.10	0.303910	1.28	**0.021514**	1.38	**0.038612**
Hsp90ab1	1.03	0.546044	1.19	**0.049921**	1.21	**0.004717**

#### 1.3 Role of CAP1 in mediating insulin sensitivity actions of resistin in BNL Cl.2 cells

Transfection of the BNL CL.2 cells with CAP1 siRNA resulted in significant change in the expression levels of 24 genes. 23 of these were upregulated (Acsl1, Adipoq, Adipor2, Apoe, Ccr4, Cebpa, Crlf2, Cs, Cxcr4, Hk2, Igflr, Ikbkb, Jak2, Lepr, Lipe, Lta4h, Mtor, Pik3r1, Ptpn1, Pycard, Serpine1, Socs3, and Stat3), and one (Irs2) was downregulated (Fig. 1A). Divided by function, these genes participate in metabolic pathways (Acsl1, Apoe1, Cebpa, Cs, Hk2, Lepr, and Lipe), insulin signaling (Igf1r, Ikbkb, Mtor, Pik3r1, Ptpn1, Socs3, and Irs2), T2DM (Adipoq, Hk2, Ikbkb, Mtor, Pik3r1, Socs3, and Irs2), adipokine signaling (Adipoq, Adipor2, Ikbkb, Jak2, Lepr, Mtor, Serpine1, Socs3, Stat3, and Irs2), innate immunity (Ikbkb, Pycard, and Irs2), inflammation (ccr4, Cxcr4, Ikbkb, Lta4h, and Pycard), apoptosis (Ikbkb, Jak2, Pycard, Serpine1, Socs3, and Irs2), or are markers of infiltrated leukocytes (Ccr4 and Crlf2) (Fig. 1, Fig. 2B).

When CAP1 siRNA-transfected cells were treated with resistin, the expression of 13 genes was significantly affected: 12 genes (Acsl1, Adipor2, Hk2, Igf1r, Ikbkb, Jak2, Lta4h, Pik3r1, Rps6kb1, Serpine1, Socs3, and Stat3) were upregulated, and 1 gene (Irs2) was downregulated (Table 3 and Fig. 2A). Divided by function, these genes were involved in metabolic pathways (Acsl1 and Hk2), insulin signaling (Igf1r, Ikbkb, Pik3r1, Rps6kb1, Socs3, and Irs2), T2DM (Hk2, Ikbkb, Pik3r1, Socs3, and Irs2), adipokine signaling (Adipor2, Ikbkb, Jak2, Serpine1, Socs3, Stat3, and Irs2), innate immunity (Ikbkb and Irs2), inflammation (Ikbkb and Lta4h), or apoptosis (Ikbkb, Jak2, Rps6kb1, Serpine1, Socs3, and Irs2) (Table 3 and Fig. 1, Fig. 2B).

## 2 Experimental design, materials and methods

### 2.1 Reagents

Mouse recombinant resistin (Sigma-Aldrich, Cat. # SRP4560) was resuspended in water to a stock concentration of 100 μg/ml and further diluted to 25 μg/ml before cell treatment.

### 2.2 Cell culture

BNL CL.2 mouse liver cells were purchased from American Type Culture Collection (ATCC) (Cat. # TIB-73) and grown in Dulbecco's Modified Eagle's Medium (DMEM) supplemented with 10% fetal bovine serum (FBS) (VWR International, Cat. # 89510–186) and antibiotic/antimycotic solution (Penicillin, Streptomycin, Amphotericin B) (Corning, Cat. # 30-004-Cl), and incubated at 37 °C with 10% CO_2_.

### 2.3 Experiment design

BNL CL.2 cells were seeded in 6-well tissue culture plates with 2 ml tissue culture medium, in a density of 0.5 × 10 [6] cells and grown for one day (to approximately 60% confluency). Resistin treatment was performed by adding 2 μl (25 μg/ml) resistin to the appropriate wells.

### 2.4 siRNA transfection

CAP1 siRNA transfection was performed using Opti-MEM Reduced Serum Medium (Gibco, Cat. # 31985–070), Lipofectamine RNAiMAX transfection reagent (Invitrogen, Cat. # 13778–075), and mouse CAP1 Silencer Select siRNA (Life Technologies, Cat. # 4390771, siRNA ID# s63297) following manufacturer's protocol. Transfection was performed for 6 hours; the cell culture medium was then replaced with complete medium for overnight cell growth.

### 2.5 RNA extraction and reverse transcription

After completing the experiments, the cells were washed one time with ice-cold PBS and RNA was extracted using TRIzol Reagent (Ambion, Cat. # 15596018), chloroform, and iso-propanol. Total RNA concentration was quantified using NanoDrop One spectrophotometer (Thermo Scientific). All of the samples were normalized to 1 mg/ml of total RNA. Reverse transcriptase (RT) reaction was performed using qScript cDNA SuperMix kit (QuantaBio, Cat. # 95048). RT reaction was performed using the following conditions: 25 °C/5 min, 42 °C/30 min, 85 °C/5 min, 4 °C/∞. After RT reaction, the cDNA samples were diluted 10 times with water.

### 2.6 Quantitative RT-PCR array

Quantitative RT-PCR array was performed using RT [2] SYBR Green ROX qPCR Mastermix (Qiagen) and RT [2] ProfilerTM PCR Array Mouse Insulin Resistance kit (QIAGEN, Cat. # PAMM-156ZA). Master mix for each plate consisted of: 1350 μl 2× RT2 SYBR Green Master Mix, 102 μl of cDNA, and 1248 μl of PCR grade water, and 25 μl of it was pipetted into each PCR plate well. Quantitative PCR was performed using the following conditions: hold stage: 50 °C/2 min, 95 °C/10 min; PCR stage: 95 °C/15 sec, 60 °C/1 min (40 cycles); melt curve stage: 60 °C/1 min, 95 °C/1 sec.

### 2.7 Data analysis

Results were obtained from 4 separate experiments. Data analysis was performed using QIAGEN web-based software (https://dataanalysis.qiagen.com). Fold change values were calculated using ΔΔCt method (2ˆ(-ΔΔCt)). p-values were calculated based on Student's t-test of the replicate 2ˆ(-ΔCt) values for each gene in the control group and the treatment groups.
